# CD8 exerts differential effects on the deployment of cytotoxic T lymphocyte effector functions

**DOI:** 10.1002/eji.200636718

**Published:** 2007-04

**Authors:** Bruno Laugel, David A Price, Anita Milicic, Andrew K Sewell

**Affiliations:** 1 Nuffield Department of Medicine, University of OxfordOxford, UK; 2 Weatherall Institute of Molecular Medicine, University of Oxford, John Radcliffe HospitalOxford, UK

**Keywords:** Antigen sensitivity, CD8 T cells, Co-receptor, Cytokines, TCR

## Abstract

Cytotoxic T lymphocytes (CTL) are equipped with a range of effector functions that contribute both to the control of intracellular pathogens and dysregulated cellular proliferation and to the development of certain immunopathologies such as autoimmune disease. Qualitative analyses of various CTL responses have revealed substantial heterogeneity in the diversity of functions that are mobilized in response to antigen. Here, we studied the influence of the CD8 co-receptor, which is known to enhance antigen recognition by CTL, on the secretion of eight different cytokines and chemokines by human CTL clones using flow cytometric bead array. Our results show that abrogation of MHC class I/CD8 interactions exerts a differential influence on the distinct individual effector functions that are elicited in response to agonist ligands. The magnitude of this co-receptor blockade inhibitory effect was clearly related to the hierarchy of cytokine secretion in terms of activation threshold because those functions requiring the highest amounts of antigen were most affected. Thus, modulation of CD8 activity can effectively tune not only the sensitivity but also the qualitative profile of CTL responses.

## Introduction

Cytotoxic T lymphocytes (CTL) are key components of the adaptive immune system, conferring protection against intracellular microbes and malignancies through the recognition of specific antigenic determinants expressed in association with major histocompatibility complex class I (MHCI) molecules on the cell surface. In addition to mediating the lysis of target cells by the directed release of cytotoxic agents upon activation 1, CTL are equipped with a range of effector functions that participate in the communication between different cellular components of the immune system and elicit anti-microbial activity independently of cytolytic mechanisms 2–4. These involve the release of soluble molecules (cytokines and chemokines) that affect the migration and cellular functions of numerous somatic cells.

Studies examining qualitative aspects of the CTL response have revealed that the arsenal of effector functions mobilized by CD8^+^ T cells in response to antigen can be strikingly heterogenous 5. Variations in the effector function profile of T cell populations have been linked to functional divergence between subsets of cells at different stages of differentiation 6, factors associated with the nature of the pathogen 7 and characteristics of the infection such as antigen dose and persistence 8, 9, the identity of the targeted epitope 10, and the expression of certain co-stimulatory molecules such as CD28 11, 12. It seems likely that, in some cases, these qualitative differences account in part for the inability of the immune system to contain certain infections or tumours. For instance, it has been proposed that the cytotoxic functions of HIV-1-specific CTL were deficient because these cells were found to produce low levels of perforin compared to CMV-specific CTL from the same donors 13, 14.

It is well accepted that the activation of different effector functions obeys a hierarchical organization, which is determined by the signal intensity elicited by T cell receptor (TCR) engagements and the level of TCR occupancy 15, 16. For example, it has been clearly established that secretion of interleukin (IL)-2 by CD4 T cells requires stronger signals than those necessary to trigger IFN-γ release 17. The activation thresholds required for these two functional outcomes, as well as for others, are thus quantitatively distinct. Therefore, the commonly described disparity between functional profiles may be explained, at least in part, by clonotypic variability with respect to antigen sensitivity. Indeed, it was recently proposed that the higher propensity of murine CTL specific for the influenza A epitope D^b^PA_224_ to elicit a diverse response, characterised by high proportions of IL-2-secreting cells, compared to D^b^NP_366_-specific cells correlated with intrinsically higher avidities for antigen 18.

Several reports have clearly established that the CTL co-receptor CD8 enhances the efficiency of antigen recognition by favouring extracellular interactions between the TCR and antigen 19, 20, and by promoting the initiation of the signalling cascade following TCR triggering 21, 22. However, this effect is not systematically required to trigger full CTL activation since ligands with high affinity for the TCR, such as can occur in the domain of xeno- and allo-reactive responses, are largely independent of co-receptor activity 23, 24. Nonetheless, most syngeneic interactions, which are more relevant to the normal context of natural immunity, have been shown to display various degrees of “CD8 dependency” 23.

Here, we investigate the influence of the CD8 co-receptor effect on the deployment of an array of effector functions by activated human CTL clones. Abrogation of co-receptor engagement using point-mutated MHCI molecules revealed that CD8 exerts a differential influence on the individual effector functions that are triggered by agonist ligands. Thus, by tuning the intensity of TCR signals, the co-receptor has a direct influence on the functional profile of CTL and thereby contributes to the qualitative consequences of antigen recognition.

## Results

### Profile and hierarchical relationship of effector functions in four CTL clones

**Figure 1:**
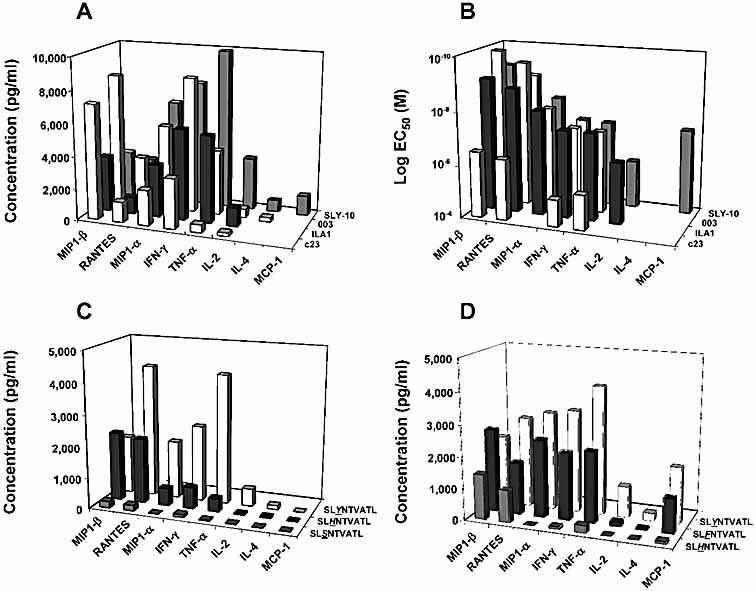
Effector function profiles and hierarchies in four human CTL clones. (A) CTL were stimulated using Hmy.2 C1R cells transfected with HLA-A2 WT molecules loaded with 10^–5^ M of the corresponding cognate peptide. The effector function profile of each clone was evaluated by measuring cytokine and chemokine concentrations in the culture supernatant after 4-h incubation. No secretion of IL-4 was observed in the case of ILA1 and c23 clones. (B) Cytokine and chemokine release by the four CTL clones was assessed in dose-response experiments using varying concentrations of antigen to pulse HLA-A2 WT Hmy.2 C1R cells. Data were plotted and fitted to a non-linear sigmoidal dose-response curve according to the following equation: Y = Y_MIN_ + (Y_MAX_ – Y_MIN_)/[1 + 10e (logEC50 – X)], and logEC50 values were calculated accordingly for the curves that reached a plateau. Effector functions logEC50 values shown for each clone are representative of three individual experiments. (C, D) Functional profiles of clones 003 (C) and SLY-10 (D) in response to three different agonist peptides; HLA-A2 WT Hmy.2 C1R cells were pulsed with 10^–5^ M of each indicated peptide prior to the assay.A degree of variability in the absolute concentration values of secreted soluble factors was observed between separate measurements. This is exemplified by the respective amounts of TNF-α and IFN-γ secreted by clone 003 in response to the index Gag p17_77–85_ epitope in (A, C). However, the order of secretion of the soluble factors with respect to peptide dose was not affected by these quantitative differences and the same hierarchy of effector functions was always observed.

The cytokine and chemokine secretion profile of four different CTL clones, described in Table 1, were examined in detail using multiplex flow cytometric bead array (Fig. 1). Stimulation with high doses of cognate antigen revealed differences in effector function diversity for each clone, with clones c23 and ILA1 showing the least diverse profiles (Fig. 1A). Surprisingly, in the case of SLY-10, substantial amounts of the CC chemokine MCP-1 were released upon activation (Fig. 1A). In addition to interclonal qualitative differences, the maximal secreted quantity of the different cytokines and chemokines seemed to follow a random pattern for each clone. In the example shown, clone SLY-10 released high amounts of TNF-α compared to MIP1-β, whereas the opposite was true in the case of clones 003 and c23 (Fig. 1A).

**Table 1:** Description of the antigen restriction and specificity of the human CTL clones used in this study

CTL clone	Origin	HLA restriction	Epitope
**c23**	HIV-1-infected patient	A*6801	ITKGLGISYGR (HIV-1 Tat_39–49_)
**SLY-10**	HIV-1-infected patient	A*0201	SLYNTVATL (HIV-1 Gag p17_77–85_)
**003**	HIV-1-infected patient	A*0201	SLYNTVATL (HIV-1 Gag p17_77–85_
**ILA1**	Healthy donor	A*0201	ILAKFLHWL (hTERT_540–548_)

The hierarchy of effector functions, established according to the logEC50 of each read-out, was identical for all tested HLA-A*0201-restricted CTL (Fig. 1B). Secretion of the chemokines MIP1-β and RANTES required the lowest amounts of antigen, whereas IL-2 release could only be detected at peptide doses that exceeded those necessary to trigger TNF-α and IFN-γ release. These results are in agreement with previous studies investigating the mobilisation of T cell functions in response to antigen 16, 17, 25. In the case of clone SLY-10, secretion of the chemokine MCP-1 required doses of antigen similar to those triggering IFN-γ and TNF-α release, two cytokines that are known to have an active role in adaptive immunity. Strikingly, the concentrations of peptide required to activate c23 were substantially higher than for the three HLA-A*0201-restricted CTL. As the affinity of the c23 TCR for cognate antigen (K_D_ =7 μM) is in the range of most antiviral TCR/pMHCI interactions measured to date (Gostick *et al*., unpublished data), this likely reflects either a low intrinsic antigen sensitivity, perhaps resulting from the impaired binding of CD8 to HLA-A*6801, or a poor loading efficiency of the unusually long 11-mer Tat_39–49_ peptide onto HLA-A*6801 molecules.

The functional profile of both HIV-1 Gag p17_77–85_-specific CTL clones in response to agonist ligands of different potencies was examined in detail (Fig. 1C, D). The 3H and 3S epitope variants have previously been identified as weak agonists of CTL clone 003 26, 27. Release of the chemokines MIP1-β and RANTES, which rank highly in the hierarchy of effector functions (Fig. 1B), remained similar in magnitude to the levels secreted in response to wild-type (WT) or ‘index’ peptide when clone 003 CTL were stimulated with the 3H variant, whereas secretion of lower-order cytokines was substantially reduced or abrogated (Fig. 1C). Stimulation with the 3S peptide triggered only minimal production of MIP1-β, RANTES and, to an even lesser extent, IFN-γ (Fig. 1C). Similarly, activation of SLY-10 with the weak agonists 3F and 3H resulted in selective blockade of lower-order effector functions (Fig. 1D).

### Co-receptor dependency is more marked for weak agonist ligands

**Figure 2:**
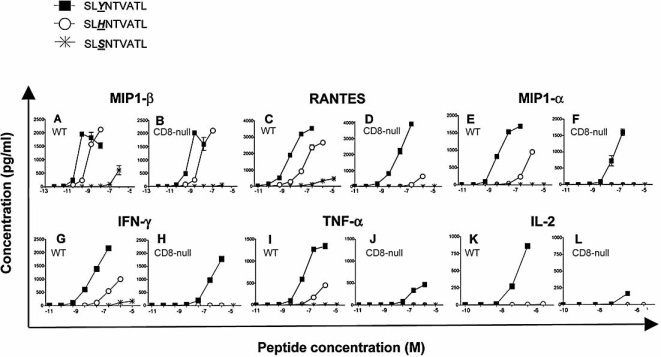
Co-receptor dependency of antigen recognition by CTL clone 003 in response to three different agonist peptides. Cytokine and chemokine production by clone 003 CTL was measured in response to three natural variants of the HLA-A2 HIV-1-derived Gag p17_77–85_ epitope presented by Hmy.2 C1R cells transfected with either HLA-A2 WT or HLA-A2 CD8-null (D227K/T228A) molecules as indicated. Concentration of each molecule was measured simultaneously from each assay well by multiplex bead array: (A, B) MIP1-β; (C, D) RANTES; (E, F) MIP1-α; (G, H) IFN-γ; (I, J) TNF-α; (K, L) IL-2. Assays were performed in duplicate; mean values and standard deviation from the mean are shown. Data shown are representative of three experiments.

**Figure 3:**
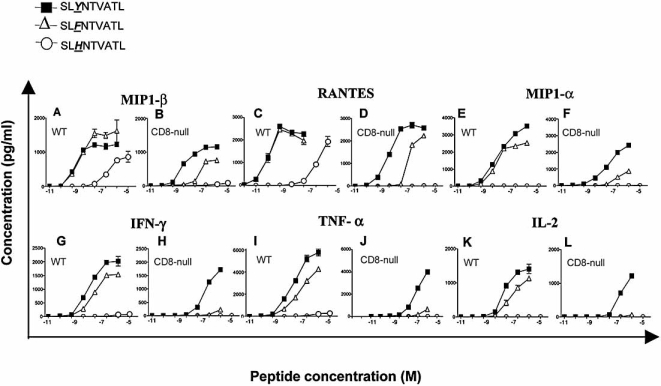
Co-receptor dependency of antigen recognition by CTL clone SLY-10 in response to three different agonist peptides. Production of soluble factors by SLY-10 CTL was measured in response to three natural variants of the HLA-A2-restricted HIV-1-derived Gag p17_77–85_ epitope presented by Hmy.2 C1R cells transfected with either HLA-A2 WT or HLA-A2 CD8-null molecules as indicated. Cytokine and chemokine concentrations were measured simultaneously from each assay well by multiplex bead array: (A, B) MIP1-β; (C, D) RANTES; (E, F) MIP1-α; (G, H) IFN-γ; (I, J) TNF-α; (K, L) IL-2. Assays were performed in duplicate; mean values and standard deviation from the mean are shown. Data shown are representative of three experiments.

The release of soluble factors by the two HLA-A*0201 HIV-1 Gag p17_77–85_-specific clones in response to naturally occurring variants with different agonistic properties was assessed in detail (Fig. 2, 3). In order to evaluate the influence of CD8 on antigen recognition, peptides were loaded on target cells expressing either WT or mutant HLA-A*0201 molecules (CD8-null) bearing a double substitution at positions 227 and 228 (D227K/T228A) of the heavy chain that abrogates binding to CD8 without affecting TCR binding 21.

For both CTL clones, recognition efficiency of the strongest ‘index’ ligand SLYNTVATL was minimally affected by the loss of the extracellular CD8 interaction with MHCI (Fig. 2, 3). In contrast, as a general observation, the recognition of weaker agonists (3H, 3F or 3S peptide variants) characterised by lower recognition efficiencies in dose-response experiments was either dramatically reduced or completely abrogated in the absence of MHCI/CD8 interactions. In the case of the 3H peptide, presentation by cells bearing CD8-null HLA-A*0201 molecules did not trigger any response even at high antigen doses for both CTL clones tested, with the exception of MIP1-β and, to a lesser extent, RANTES release (Fig. 2A–D; 3A–D). Similar observations held true for recognition of the 3F peptide by SLY-10 for which MIP1-β, RANTES and MIP1-α release were reduced by the CD8-null mutation (Fig. 3A–F), whereas no IFN-γ, TNF-α or IL-2 were produced in the obsence of MHCI/CD8 interaction (Fig. 3G–L). Thus, these results indicate that CD8 dependency is inversely correlated with the potency of the agonist ligand, a notion consistent with data reported in other studies 23.

### Correlation between effector function hierarchy and susceptibility to co-receptor blockade

Two observations can be made from the dose-response secretion patterns obtained from CTL clones 003 and SLY-10. First, all functional outcomes resulting from antigen exposure are not equally affected by disruption of the MHCI/CD8 interaction. This is exemplified in the case of clone 003 stimulated with the 3H peptide variant by comparing the pattern of MIP1-β release (Fig. 2A, B), which was only reduced to a small extent in the case of the CD8-null targets, with those for MIP1-α, IFN-γ and TNF-α (Fig. 2E–J), in which cases MHCI/CD8 disruption abrogated secretion. Similar observations were made in the case of clones 003 and SLY-10 stimulated with all tested HIV-1 Gag p17_77–85_ peptide variants (Fig. 2, 3).

Second, the main factor governing this differential co-receptor dependency appears to be the hierarchy of effector functions. Of all tested effector functions, MIP1-β secretion was consistently the least CD8-dependent in addition to being the function triggered at the lowest level of antigen (Fig. 1B; 2A; 3A). IL-2 secretion, in contrast, occupies the opposite end of the functional spectrum, requiring the highest amounts of antigen (Fig. 1B; 2K; 3K). Accordingly, for most agonist ligands tested, faithful MHCI/CD8 interactions were required to trigger efficient IL-2 release. The secretion profiles of IFN-γ and MIP1-α oscillated between low and total CD8 co-receptor dependency, according to the recognition efficiency characteristics of the ligand under consideration. Altogether these results point to a crucial role for both antigen potency and effector function hierarchy in determining the degree of co-receptor dependency of antigenic stimulation.

### Atypical secretion of the chemokine MCP-1 and co-receptor dependency

**Figure 4:**
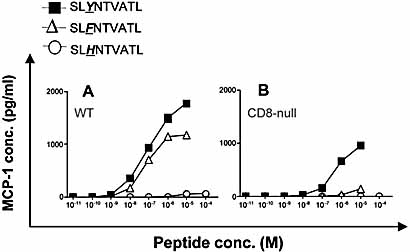
Dose-response profile and co-receptor dependency of MCP-1 secretion by CTL clone SLY-10. MCP-1 levels in the supernatant of assay wells containing CTL mixed with APC expressing HLA-A2 WT (A) or HLA-A2 CD8-null (B) molecules were determined from the same assay well shown in Fig. 3. Mean values and standard deviation from the mean are shown.

Secretion of the CC chemokine MCP-1 by CTL clone SLY-10 appeared to be an intriguing feature since, to the best of our knowledge, secretion of this molecule by T cells has not been documented previously. Initial dose-response experiments revealed that the secretion profile of MCP-1 had a logEC50 nearly identical to those for TNF-α and IFN-γ (Fig. 1B). In agreement with the notion that the hierarchical organization of CTL effector functions is an important factor that determines the influence of co-receptor interactions on CTL activation, MCP-1 release showed a pattern of CD8 dependency similar to that of TNF-α and IFN-γ (Fig. 3G–J; Fig. 4). Thus, it seems that secretion of MCP-1 by SLY-10 both requires relatively high amounts of antigen and displays a degree of co-receptor dependency similar to that of two cytokines known to exert antiviral functions *in vivo*.

## Discussion

The range of effector functions deployed by CTL in response to antigen *in vivo* is known to exhibit a degree of variability. Several factors, including intrinsic cellular properties related to differentiation status and interclonal functional differences, have been shown to have an impact on qualitative aspects of the CTL response. Secretion of the chemokine MCP-1 (CCL2) by one of the CTL clones used in our study illustrates this type of inherent functional diversity. Release of MCP-1 has been described for a variety of cell types including dendritic cells, endothelial cells, fibroblasts and macrophages 28. This chemokine is involved in promoting the recruitment of monocytes to infection sites and has also been proposed to play a role in CD4 T cell Th1/Th2 polarization 29 by negatively regulating the Th1 response 30. Secretion of MCP-1 was shown to be induced by different sorts of stimuli including the action of several cytokines such as IFN-γ, IL-1, IL-4 and TNF-α 31, 32 as well as vascular endothelial stress 33. Important up-regulation of MCP-1 serum levels was notably shown to occur in atherosclerotic lesions 34. Our results suggest that antigenic activation of CTL may be another source of MCP-1 *in vivo*. By extrapolation of the results obtained with clone SLY-10, MCP-1 is likely to be triggered by levels of antigen characteristic of *in vivo* viral infection settings since its position within the functional hierarchy was similar to that of IFN-γ and TNF-α. It is possible that the secretion of MCP-1 by our CTL clone is the exception rather than the rule and further work will be required in order to establish whether this observation is of general relevance.

Antigen load 35 and the nature of antigenic stimulation 8 have also been proposed to affect the quality of T cell responses. It is well established that stimulation with suboptimal altered peptide ligands can induce only some of the effector functions in the T cell armamentarium. Weak agonist ligands only elicit those functions that normally require low doses of strong agonists while a more intriguing category of antigens, the so-called partial agonists, selectively elicit a set of functional outcomes irrespective of the established functional hierarchy 36, 37. In our system, the peptide variants we studied all belonged to the former category since suboptimal antigens triggered only the secretion of effector functions at the top of the functional hierarchy. Thus, the correlation between ligand potency, response diversity and functional hierarchy remained apparent in all cases; for peptides of intermediate potency, only secretion of IL-2 was lost, whereas activation with lower potency agonists resulted in the additional abrogation of IFN-γ, TNF-α and MIP1-α (as well as MCP-1 in the case of SLY-10).

**Figure 5:**
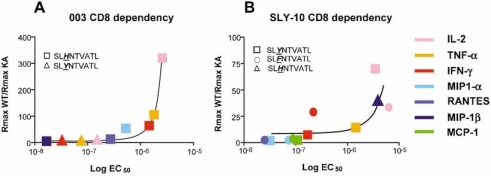
The dependency on CD8 co-receptor activity for the efficient secretion of each effector function varies as an inverse function of antigen sensitivity. Each cytokine is colour-coded as indicated and ranked on the x axis according to the peptide concentration (M) required to trigger half-maximum release (logEC50) in response to a particular ligand (represented by different geometrical figures): 3Y and 3H for clone 003 (A), and 3Y, 3F and 3H for clone SLY-10 (B). Only those functions for which the dose-response profile levelled off, yielding reliable EC50 values by curve fitting, were taken into account to plot this graph. Functions with low EC50 values (<10^–8^ M) were excluded for clarity. “Co-receptor dependency” for each read-out is represented as the ratio between maximal cytokine or chemokine concentration (R_MAX_) measured for each ligand presented in the context of WT HLA-A2 and CD8-null (KA) HLA-A2 molecules. High R_MAX_WT/R_MAX_KA values are thus indicative of a high degree of CD8 dependency. In contrast, effector functions for which the co-receptor interaction has little effect have R_MAX_WT/R_MAX_KA ratio values close to 1. R_MAX_WT/R_MAX_KA ratios for functional read-outs with EC50 values below 10^–8^ M were close to 1 (data not shown). The ratio between maximum responses triggered by WT and CD8-null HLA-A2 molecules increases exponentially for logEC50 values >10^–7^–10^–6^ M. Data were fitted to the equation Y = 1 × exp(K × X); K = 1.45×10^6^ (A); K = 0.38×10^6^ (B).

Our data reveal that the lower-order CTL effector functions, *i.e.* those requiring high amounts of antigen, are disproportionately affected by co-receptor blockade. Indeed, there appears to be an absolute antigen sensitivity threshold below which disruption of CD8 binding dramatically impairs the recruitment of any effector function, regardless of the potency of the agonist ligand considered. Specifically, in the context of the two HIV-1 Gag p17-specific CTL clones 003 and SLY-10, effector functions for which half-maximal responses were elicited at peptide concentrations above 10^–7^–10^–6^ M showed a high degree of co-receptor dependency (Fig. 5). Consequently, the main difference between a strong and a weak agonist was the number of functions impaired by abrogation of co-receptor binding. For instance, only the secretion of lower-order factors such as IL-2 was substantially affected in the case of stimulation with potent antigens while most, if not all, effector functions triggered in response to the weakest agonists relied on faithful MHCI/CD8 interactions even at high antigen density.

The different co-receptor activities of CD8 are known to synergize to enhance proximal signalling events 21, 38, which are crucial for increasing the sensitivity of antigen recognition. Our results indicate that co-receptor-mediated tuning of the signalling pathways downstream of TCR engagement not only improves the dose-response pattern of CTL activation but also partially determines the diversity of effector functions mobilised at a given dose of antigen. As a consequence, we predict that the cytokine profile of a CTL response is substantially influenced by co-receptor engagement for the natural antigen densities present on the surface of virally infected cells. This issue becomes particularly salient in the case of suboptimal weak agonist ligands such as the natural HIV-1 altered peptide ligands used here. The effect of the co-receptor on the quality of the CTL response reported here is also likely to be an important determinant in the context of tumor-specific and autoimmune CTL responses. The TCR/pMHCI affinities of these types of interactions are believed to occupy the lower reaches of the functionally relevant TCR/pMHCI affinity spectrum, a situation in which the contribution of CD8 to CTL activation is known to be crucial 23.

Recent investigations of CTL functional profiles in mice and humans have lead to the suggestion that the functional diversity of CTL responses provides an indication of immune antiviral control and clinical outcome 35. Specifically, the CTL responses to viral epitopes measured *ex vivo* from vaccinated individuals were shown to be more diverse than in the context of natural immunization 7. Moreover, it has recently been established in the context of HIV-1 infection that the proportion of CTL exhibiting a polyfunctional response to multiple epitopes was substantially higher in long-term nonprogressors than in infected individuals with a less favourable clinical course 39. The authors of this report proposed that the functional diversity of the CTL response, rather than its magnitude, provides an indication of antiviral protection in HIV-1-infected patients; this might also hold true in the context of other viral infections. Interestingly, the two cytokines consistently present at a higher frequency in antigen-specific CTL from long-term nonprogressors were the lower-order effector functions TNF-α and IL-2.

Recent reports have suggested a possible regulatory role for CD8 with respect to the responsiveness and sensitivity of CTL mediated by down-regulation of expression 40, 41, modifications of TCR-CD8 colocalization 42 and post-translational modulation of CD8 activity at different developmental stages 43, 44. Our results indicate that if such regulatory activity is indeed a means to modulate the state of CTL responsiveness, then interference with CD8 co-receptor activity is also likely to tune qualitative aspects of the response to antigen by disproportionately affecting lower-order effector functions. Whether this phenomenon has functional implications *in vivo* is difficult to assess, but it is tempting to speculate that down-regulation of CD8 co-receptor activity, such as appears to occur following antigenic stimulation of effector T cells in particular 41, 45, might serve as a regulatory feedback mechanism that tames the CTL response in a relevant spatio-temporal context. According to our data, one consequence of such a mechanism would be the selective inhibition of lower-order CTL functions (IFN-γ, TNF-α and IL-2 especially) upon antigen re-exposure in the short term without major effects on higher-order effector mechanisms such as cytolysis and the release of MIP1-β and RANTES. It is interesting to note that release of the cytokines IFN-γ and TNF-α, which have been implicated as causal agents in septic shock 46, is highly dependent on CD8. Even though many other immune cells, including effectors of innate immunity, are major sources of these molecules, it has been shown that inadequate cytokine release by CTL was sufficient to induce severe consequences following viral infections 47. Tight regulation of the stimulatory mechanisms inducing cytokine and chemokine release is therefore required in order to keep the immune response under control.

A recent study investigating the role of CD8 in the activation of CTL by soluble tetrameric pMHCI complexes suggested that, in contrast to naive cells, cytokine release by memory CTL did not require co-receptor interactions whereas calcium mobilization and proliferation were CD8-dependent for both naive effector and memory cells 48. This is in agreement with the notion that elicitation of some effector functions differentially requires co-receptor interactions. The cells we used in this study were CTL clones established after several rounds of *in vitro* stimulation and it is not clear to what extent the memory phenotype of these cells is meaningful. Yet, these CTL are clearly antigen-experienced and exhibit differential degrees of co-receptor dependency for the release of cytokines. The apparent minor discrepancy between our results and those of Kerry and colleagues 48 with regard to the CD8 dependency of cytokine release may lie in the fact that we used cell surface presentation for antigen-specific stimulation and that, in our study, differences in the release of effector molecules were most obvious at low cell surface antigen densities, a situation very different from activation with soluble multimeric antigens.

Finally, it was reported that blocking the interaction between CD4 and MHC class II molecules differentially affected the secretion of IL-2 *versus* IL-3 cytokines by CD4^+^ T cells 49. This suggests that regulation of the response to antigen by modulation of co-receptor activities may be a general feature of T cells and that a *raison d'être* of the dual TCR/co-receptor antigenic ligand recognition system may be to regulate the T cell response at the level of the incoming stimulus through differential modulation of co-receptor activity. This phenomenon could potentially contribute to the establishment of T cell tolerance in the periphery. Furthermore, from a practical perspective, the effect of CD8 on CTL activation could potentially be exploited therapeutically to down-modulate deleterious CTL responses.

## Materials and methods

### Cytokine and chemokine bead arrays

Approximately 10 000 APC were pre-pulsed with the indicated concentrations of peptide and washed twice with serum-free RPMI 1640 medium (Sigma) supplemented with 2 mM glutamine (Gibco), 100 U/mL penicillin (Gibco) and 100 U/mL streptomycin (Gibco) (RPMI-PSG medium). CTL (15 000 or 30 000) were added in each assay well and incubated for 4 h at 37°C in 96-well plates. Cells were pelleted by centrifugation; supernatants were harvested and assayed with the human Th1/Th2 Cytokine and Chemokine kits (BD Pharmingen) according to the manufacturer's instructions. Analysis was performed with a FACSCalibur (Becton Dickinson) flow cytometer.

### B cell lines

EBV-immortalized Hmy2.C1R cells expressing WT HLA-A*0201 or CD8-null (D227K/T228A) HLA-A2 molecules are described elsewhere 21. These cells were maintained in RPMI-PSG medium supplemented with 10% heat-inactivated fetal calf serum (Globepharm) (R10 medium). Medium was replaced every 2 days to keep the cells in a state of constant growth.

### Generation of CTL clones

Peripheral blood mononuclear cells isolated from HLA-A*0201 positive healthy donors or patients were stimulated with antigen by autologous presentation of peptide diluted at a final concentration of 10^–6^ or 10^–7^ M. Cells were maintained in R10 medium to which IL-2 (Peprotech) was gradually added from day 3 post-stimulation up to a maximum concentration of 100 U/mL. Following successful expansion of antigen-specific cells, CTL clones were isolated by limiting dilution in a 96-well plate at an average of 0.3 cells per well containing R10 supplemented with 100 U/mL IL-2 (Peprotech), 10% T-stim (Becton Dickinson) and mixed irradiated allogeneic feeder cells from at least three unrelated donors stimulated with phytohemagglutinin. The antigen specificity of growing cells was tested by pMHCI multimer staining and IFN-γ ELISPOT assays.

### Peptides

The hTERT_540–548_ (ILAKFLHWL) peptide was purchased from Pepscan (Lelystad, The Netherlands). The HIV-1 Tat_39–49_ HLA-A*6801-restricted peptide (ITKGLGISYGR) together with the HIV-1 Gag p17_77–85_ index peptide (SLYNTVATL) and mono-substituted variants thereof were purchased from Invitrogen. Peptide preparations used in this study were purified by mass spectrometry and were >95% pure. Powder was initially dissolved in DMSO and further diluted in serum-free RPMI-PSG to the desired concentrations.

## Note added in proof

The publication details of the manuscript referred to as “unpublished data” in the text and describing the biophysical properties of the TCR of CTL clone c23 are:

## Abbreviation:

MHCI: MHC class I
